# Atypical femoral fracture in a patient with nutritional osteomalacia: a case report and literature review

**DOI:** 10.1093/jscr/rjaf020

**Published:** 2025-01-22

**Authors:** Mohammed Alshehri, Abdulrahman Alzahrani, Abdulrahman Aljehani, Abdullah Saeed, Ziad Aljaafri

**Affiliations:** Department of Orthopedic Surgery, Ministry of the National Guard – Health Affairs, Riyadh, Saudi Arabia; Department of Orthopedic Surgery, Ministry of the National Guard – Health Affairs, Riyadh, Saudi Arabia; Department of Orthopedic Surgery, Ministry of the National Guard – Health Affairs, Riyadh, Saudi Arabia; Department of Orthopedic Surgery, Ministry of the National Guard – Health Affairs, Riyadh, Saudi Arabia; Department of Orthopedic Surgery, Ministry of the National Guard – Health Affairs, Riyadh, Saudi Arabia; College of Medicine, King Saud bin Abdulaziz University for Health Sciences, Riyadh, Saudi Arabia

**Keywords:** case report, atypical femoral fracture, AFF, nutritional osteomalacia

## Abstract

Atypical femoral fractures (AFF) are rare stress fractures with specific diagnostic criteria, as outlined in a report published by the American Society for Bone and Mineral Research. These criteria are categorized into major and minor features, and AFF can be classified as either complete or incomplete. Bisphosphonates have been shown to increase the risk of AFF, and most cases of AFF are associated with bisphosphonate use. We present a unique case of AFF in a young woman with no history of bisphosphonate use. She was taking oral contraceptive pills and inhaled corticosteroids for asthma. Later, she was diagnosed with nutritional osteomalacia. The patient was managed surgically with bilateral intramedullary nailing, resulting in a favorable outcome.

## Introduction

Atypical femoral fractures (AFF) are rare stress fractures with specific diagnostic criteria, as outlined in a report by the American Society for Bone and Mineral Research [1]. These criteria are divided into major and minor features, and AFF can be classified as either complete or incomplete. To classify a fracture as an AFF, four out of five major criteria must be met. The major features include: (i) the fracture occurring anywhere along the femur, from just distal to the lesser trochanter to just proximal to the supracondylar flare, (ii) minimal or no trauma, such as a fall from standing height or less, (iii) a transverse or short oblique fracture pattern, (iv) the fracture being non-comminuted, and (v) complete fractures involving both cortices, potentially with a medial spike, while incomplete fractures involve only the lateral cortex. Minor features include: (i) localized periosteal reaction of the lateral cortex, (ii) generalized increase in cortical thickness of the diaphysis, (iii) prodromal symptoms, such as dull or aching pain in the groin or thigh, (iv) bilateral fractures or symptoms (v) delayed healing, (vi) comorbid conditions, and (vii) use of pharmaceutical agents [1, 2]. As reported in the literature, bisphosphonate use has been shown to increase the risk of AFF [3–5].

A review of the current literature reveals only a few reported cases of AFF in patients with underlying nutritional osteomalacia. This study presents a unique case of AFF in a woman, where the fracture was the first presenting symptom that led to the diagnosis of osteomalacia. The patient was managed operatively with bilateral intramedullary nailing, resulting in a positive outcome.

## Case report

A 38-year-old woman, known to have obesity with a body mass index of 38 and asthma, treated with inhaled fluticasone and salbutamol, presented to the emergency department after falling on her right side. She tripped and landed on her right side, with her primary complaint being right thigh pain. Upon evaluation, she denied any history of head trauma or loss of consciousness, and there was no history of previous thigh pain or contralateral thigh pain.

**Figure 1:**
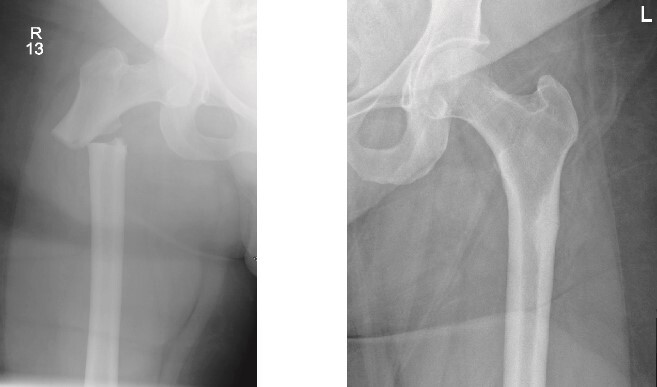
Anteroposterior (AP) view of the right and left femurs pre-operative. With ismuths of 7.7 in R and 6.9 in L

On examination, the patient was alert, oriented, and vitally stable. There were no open wounds on the right thigh, but mild tenderness was noted. The soft compartments were palpable, and the ankle range of motion was full. Distal neurovascular structures were intact. The left thigh showed no wounds or tenderness, and the hip range of motion was 0° to 60°, limited by pain in the right hip, as reported by the patient. Distal neurovascular structures were also intact on the left side. X-rays of both the right and left femurs, taken in the emergency department, are shown in Fig. 1.

Surprisingly, as seen in the X-rays in Fig. 1, the patient had a right subtrochanteric femur fracture and a stress line in the contralateral femoral cortex. While preparing and optimizing the patient for surgery, the endocrinology team was consulted regarding this presentation. According to their assessment, the patient’s laboratory findings were reassuring, and they confirmed that we could proceed with the surgical plan. Bone mineral density (BMD) testing was planned for afterward, and the endocrinology team would review the results. Surgery was scheduled for the following day.

Our surgical plan involved closed reduction and internal fixation (CRIF) of the right subtrochanteric femur fracture with an intramedullary (IM) nail, and prophylactic placement of an IM nail in the left femur. The patient was brought to the operating room and positioned on the fracture table under general anesthesia. The procedure began with the right femur after the usual sterile draping and prepping. A 3-centimeter incision was made proximal to the greater trochanter. A guidewire was inserted after identifying the entry site with portable fluoroscopy. The guidewire was advanced, followed using a proximal entry reamer. The fracture was then reduced using fracture traction and manual manipulation. A K-wire was advanced to secure the reduction. The appropriate nail size was measured, and reaming was performed. For the right femur, reaming was done up to size 12. The IMN was then inserted under fluoroscopic guidance. Two recon screws (90 mm) were placed on the right side, followed by two distal screws. Fracture compression was achieved by back-hammering the nail. Following fixation, the wound was irrigated, and closure was performed with Vicryl sutures for the subcutaneous tissue and clips for the skin.

**Figure 2:**
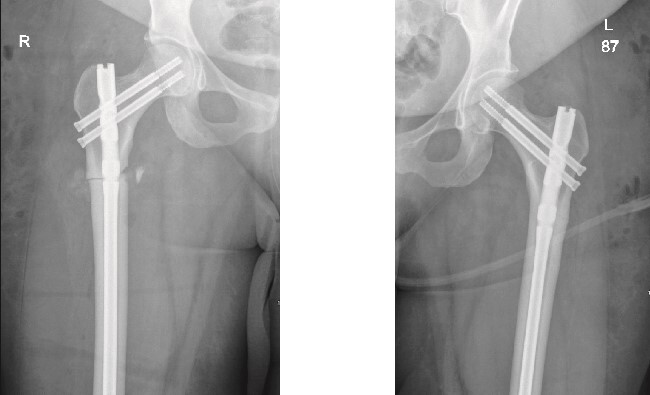
Anteroposterior (AP) view of the right and left femurs after CRIF with IM nail.

The left side was then prepped and draped. The same procedure was performed, with the nail size changed to 9 mm x 360 mm. Two proximal recon screws (90 mm) were inserted, followed by two distal screws. After fixation, the wound was irrigated, and closure was completed using Vicryl sutures for the subcutaneous tissue. The skin was closed with clips, and a sterile dressing was applied. The patient was extubated and transferred to her bed in stable condition, then moved to the post anesthesia care unit in stable condition. Immediate post-operative X-rays of both the right and left femurs are shown in Fig. 2.

The patient was kept under observation and had an uneventful hospital stay, with daily rounds and dressing checks, in addition to physiotherapy sessions. On postoperative Day 3, the pressure dressings were removed from both wounds. The primary dressings were found to be intact and dry. During the hospital stay, the clinical nutritionist was consulted to assess and optimize the patient’s condition. By postoperative Day 8, the patient was deemed fit for discharge after meeting the physiotherapy goals. The patient was then followed up in the orthopedic trauma clinic and by the endocrinology team, as previously recommended during the admission.

**Figure 3:**
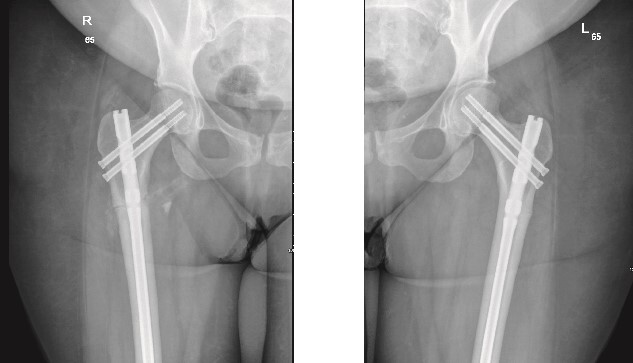
6-weeks follow-up anteroposterior (AP) view of the right and left femurs after CRIF with IM nail.

**Figure 4:**
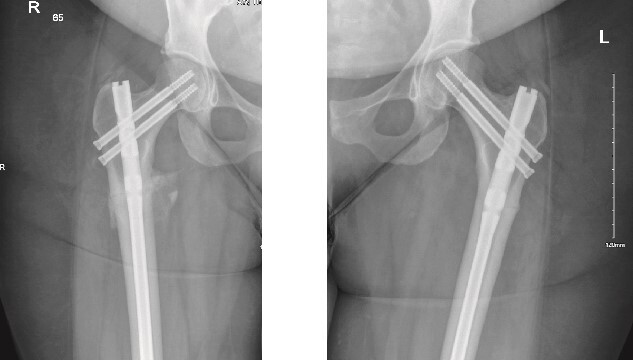
6-months follow-up anteroposterior (AP) view of the right and left femurs after CRIF with IM nail.

The patient was seen in the clinic at 2 weeks, 6 weeks, and 6 months post-surgery. She was doing well, reporting only mild pain. She was fully weight-bearing with the assistance of a cane and had good range of motion (Figs 3 and 4).

As part of the follow-up, the endocrinology team continued to monitor the patient for further investigation into the possible causes of the fractures. A BMD scan was performed and showed normal results. Based on their assessment, the endocrinology team diagnosed the patient with osteomalacia, most likely of nutritional origin.

## Discussion

AFF are considered rare, but as previously reported in the literature, they have been observed in patients with a prolonged history of bisphosphonate use, particularly in the aging population [3–5].

When classifying AFF as complete or incomplete, this distinction helps guide the treatment plan. In cases of complete fractures, fixation with a cephalomedullary intramedullary nail (IMN) spanning the entire femur is typically recommended, as previously discussed [1, 6]. In our case, the right femur was classified as a complete AFF, and the treatment plan involved closed reduction and internal fixation with a long cephalomedullary nail.

On the contralateral side, the fracture was a cortical stress line, and a prophylactic nailing was performed. As reported in the literature, the most common approach for incomplete AFFs is surgical fixation, as these fractures are more likely to progress to complete fractures [7]. Previously reported cases managed prophylactically have shown full radiographic union, with patients being pain-free ~8 months post-surgery [8]. In contrast, our patient achieved full clinical union just 2 months postoperatively, mobilizing independently with a cane for support and being pain-free.

According to the endocrinology team, the patient was diagnosed with nutritional osteomalacia. One study in the literature reported a similar case in which a young woman presented with bilateral femoral shaft fractures. She was treated with intramedullary nailing and achieved union within three months [9]. Other studies have also documented similar presentations in patients with nutritional osteomalacia [10, 11].

This highlights the importance of a multidisciplinary approach in managing such cases, where fractures may be the first sign of nutritional osteomalacia. Proper management of the underlying condition can lead to excellent outcomes for the patients.

## Conclusion

AFF are an uncommon stress fractures that should be considered even in young patients not using bisphosphonates, especially those presenting with prodromal symptoms and no history of trauma. Once AFF is diagnosed the contralateral side should be checked, the option to is to go for close surveillance or prophylactic fixation based on the patient’s clinical condition and radiological findings.

## References

[1] Shane E , Burr D, Abrahamsen B, et al. Atypical subtrochanteric and diaphyseal femoral fractures: second report of a task force of the American Society for Bone and Mineral Research. J Bone Miner Res 2014;29:1–23. 10.1002/jbmr.1998. 23712442

[2] Tile L , Cheung AM. Atypical femur fractures: current understanding and approach to management. Ther Adv Musculoskelet Dis 2020;12:1759720X20916983. 10.1177/1759720X20916983. PMC7443989 32913448

[3] Qiu S , Dhaliwal R, Divine G, et al. Differences in bone histomorphometry between white postmenopausal women with and without atypical femoral fracture after long-term bisphosphonate therapy. J Bone Miner Res 2024;39:417–24. 10.1093/jbmr/zjae018. 38477744 PMC11262150

[4] Lo JC , Grimsrud CD, Ott SM, et al. Atypical femur fracture incidence in women increases with duration of bisphosphonate exposure. Osteoporos Int 2019;30:2515–20. 10.1007/s00198-019-05112-5. 31555883 PMC7449240

[5] Starr J , Tay YKD, Shane E. Current understanding of epidemiology, pathophysiology, and management of atypical femur fractures. Curr Osteoporos Rep 2018;16:519–29. 10.1007/s11914-018-0464-6. 29951870 PMC6061199

[6] Park YC , Song HK, Zheng XL, et al. Intramedullary nailing for atypical femoral fracture with excessive anterolateral bowing. J Bone Joint Surg Am 2017;99:726–35. 10.2106/JBJS.16.00760. 28463916

[7] Ha YC , Cho MR, Park KH, et al. Is surgery necessary for femoral insufficiency fractures after long-term bisphosphonate therapy? Clin Orthop Relat Res 2010;468:3393–8. 10.1007/s11999-010-1583-2. 20865463 PMC2974881

[8] Egol KA , Park JH, Prensky C, et al. Surgical treatment improves clinical and functional outcomes for patients who sustain incomplete bisphosphonate-related femur fractures. J Orthop Trauma 2013;27:331–5. 10.1097/BOT.0b013e31827240ae. 22986315

[9] Khan AR , Rehan F, Nasrumminallah M, et al. Bilateral femoral shaft fractures in osteomalacia: a case report and literature review. Int J Surg Case Rep 2024;123:110302. 10.1016/j.ijscr.2024.110302. 39288487 PMC11420458

[10] Aaron JE , Gallagher JC, Anderson J, et al. Frequency of osteomalacia and osteoporosis in fractures of the proximal femur. Lancet 1974;1:229–33. 10.1016/s0140-6736(74)92545-8. 4130245

[11] Garg S , Singh J, Bahadur R, et al. Nutritional osteomalacia-induced bilateral neck femur fracture in an elderly patient: a case report. J Orthop Case Rep 2020;10:19–22. 10.13107/jocr.2020.v10.i08.1840. PMC7933646 33708703

